# Al_2_O_3_ Nanoparticle Addition to Commercial Magnesium Alloys: Multiple Beneficial Effects

**DOI:** 10.3390/nano2020147

**Published:** 2012-05-29

**Authors:** Muralidharan Paramsothy, Jimmy Chan, Richard Kwok, Manoj Gupta

**Affiliations:** 1 Department of Mechanical Engineering, National University of Singapore, 9 Engineering Drive 1, 117576, Singapore; Email: mpegm@nus.edu.sg; 2 Singapore Technologies Kinetics Ltd (ST Kinetics), 249 Jalan Boon Lay, 619523, Singapore; Email: chanjimmy@stengg.com (J.C.); kwokrichard@stengg.com (R.K.)

**Keywords:** Al_2_O_3_ nanoparticles, AZ series, ZK series, microstructure, mechanical properties

## Abstract

The multiple beneficial effects of Al_2_O_3_ nanoparticle addition to cast magnesium based systems (followed by extrusion) were investigated, constituting either: (a) enhanced strength; or (b) simultaneously enhanced strength and ductility of the corresponding magnesium alloys. AZ31 and ZK60A nanocomposites containing Al_2_O_3_ nanoparticle reinforcement were each fabricated using solidification processing followed by hot extrusion. Compared to monolithic AZ31 (tension levels), the corresponding nanocomposite exhibited higher yield strength (0.2% tensile yield strength (TYS)), ultimate strength (UTS), failure strain and work of fracture (WOF) (+19%, +21%, +113% and +162%, respectively). Compared to monolithic AZ31 (compression levels), the corresponding nanocomposite exhibited higher yield strength (0.2% compressive yield strength (CYS)) and ultimate strength (UCS), lower failure strain and higher WOF (+5%, +5%, −4% and +11%, respectively). Compared to monolithic ZK60A (tension levels), the corresponding nanocomposite exhibited lower 0.2% TYS and higher UTS, failure strain and WOF (−4%, +13%, +170% and +200%, respectively). Compared to monolithic ZK60A (compression levels), the corresponding nanocomposite exhibited lower 0.2% CYS and higher UCS, failure strain and WOF (−10%, +7%, +15% and +26%, respectively). The capability of Al_2_O_3_ nanoparticles to enhance the properties of cast magnesium alloys in a way never seen before with micron length scale reinforcements is clearly demonstrated.

## 1. Introduction

Magnesium is actively used as a lightweight metal in the aerospace and automotive industries. It is 35% lighter than aluminum and 78% lighter than steel. Its use ensures lower fuel consumption, reduced CO_2_ emissions and thus a greener earth. Magnesium is also used as a lightweight metal in the consumer electronics and sports industries, replacing plastics. Here, the superior electromagnetic shielding and vibration damping characteristics of magnesium contribute to better health and well-being of the human body. This also leads to better quality of life globally. AZ31 is a very commonly used Al-containing (or Zr-free) Mg alloy and is characterized by: (a) low cost; (b) ease of handling; (c) good strength and ductility; and (d) resistance to atmospheric corrosion. AZ31 has recently been surface-reinforced with SiC microparticulates [1], C60 molecules [2], and multi-walled carbon nanotubes [3], using the friction stir processing technique. In these studies, good dispersion and hardening of the base matrix were reported. ZK60A (Mg-Zn-Zr system) is a commonly used Zr-containing (or Al-free) Mg alloy and is characterized by: (a) high strength and ductility after aging (T5 heat treatment); (b) good creep resistance; (c) poor arc weldability due to hot-shortness cracking; and (d) excellent resistance weldability. Superplasticity of the Mg-Zn-Zr system has been recently studied [4,5,6]. Here, superplasticity was attributed to fine grain size (lesser twinning effects) and crystallographic textural effects. Similarly, the superplasticity of Mg-Zn-Zr system reinforced with SiC particles of micron or sub-micron size has also been reported [7,8,9]. Regarding aluminum borate whiskers, the composite interface formed with the Mg-Zn-Zr alloy matrix has also been studied and improved [10]. The fatigue related effects of Al_2_O_3_ nanoparticle addition to AZ series magnesium alloy have been studied in detail [11]. Additionally, the strength related effects of *in situ* formed nanoparticles in magnesium have also been reviewed [12]. In recent years, three methods that have been tried to improve the strength, ductility and modulus of Mg are: (a) use of various oxide nanoparticles for improving strength and ductility [13,14,15]; (b) use of metallic particles such as Ti and Mo for improving ductility [16,17,18]; and (c) use of micron size ceramic particulates for improving strength and modulus [19,20]. An open literature search has revealed that limited attempts have been made to simultaneously increase strength and ductility (tensile as well as compressive) of magnesium alloys with Al_2_O_3_ nanoparticles using a high volume production spray-deposition based solidification processing technique.

Accordingly, the primary aim of this study was to simultaneously increase strength and ductility (tensile as well as compressive) of AZ31 and ZK60A with Al_2_O_3 _ nanoparticles. Disintegrated melt deposition followed by hot extrusion was used to synthesize the AZ31/Al_2_O_3_ and ZK60A/Al_2_O_3 _nanocomposites.

## 2. Results and Discussion

### 2.1. Synthesis of Monolithic AZ31, ZK60A and Derived Nanocomposites

No macropores or shrinkage cavities were observed in the cast monolithic and nanocomposite materials. No macrostructural defects were observed for extruded rods of monolithic and nanocomposite materials. Synthesis of monolithic and nanocomposite materials—the final form being extruded rods—was successfully accomplished with: (a) no detectable metal oxidation; (b) no detectable reaction between the graphite crucible and melts. The inert atmosphere used during disintegrated melt deposition (DMD) was effective in preventing oxidation of the Mg melt. No stable carbides of Mg or Al formed due to reaction with the graphite crucible.

### 2.2. Microstructural Characteristics

Microstructural characterization of extruded samples is discussed in terms of: (a) grain size; (b) Al_2_O_3_ nanoparticle reinforcement characteristics; and (c) crystallographic texture.

**Figure 1:**
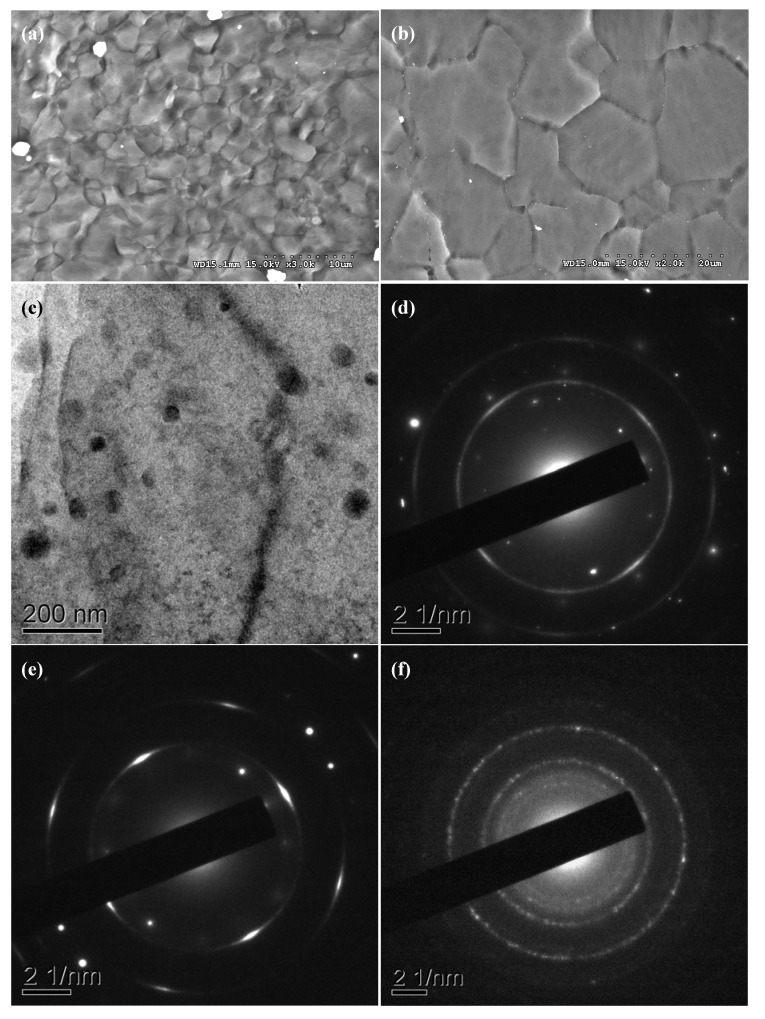
Representative SEM micrographs showing grain size in: (**a**) monolithic AZ31 and derived nanocomposite; and (**b**) monolithic ZK60A and derived nanocomposite; (**c**) representative TEM micrograph showing the presence of individual nanoparticles and fine intermetallic particles in each nanocomposite; representative selective area electron diffraction (SAED) diffraction patterns of: (**d**) AZ31/Al_2_O_3_ nanocomposite; (**e**) ZK60A/Al_2_O_3_ nanocomposite; and (**f**) Al_2_O_3_ nanoparticles. Linked with Table 2.

Microstructural analysis results revealed that there was no significant change in grain size for each nanocomposite compared to the corresponding monolithic alloy as shown in Table 1 and Figure 1. This suggested the inability of Al_2_O_3_ nanoparticles to serve as either nucleation sites or obstacles to grain growth during solid state cooling. Nearly equiaxed grains were observed in each monolithic material and derived nanocomposite.

**Table 1:** Results of grain characteristics and microhardness of AZ31, ZK60A and derived nanocomposites.

Material	Al_2_O_3_ (vol.%)	Grain characteristics ^a^		Microhardness (HV)
		Size (μm)	Aspect ratio	
AZ31	–	4.0 ± 0.9	1.4	64 ± 4
AZ31/1.5 vol.% Al_2_O_3_	1.50	2.3 ± 0.7	1.6	83 ± 5 (+30) ^b^
ZK60A	–	11.1 ± 3.0	1.5	117 ± 6
ZK60A/1.5 vol.% Al_2_O_3_	1.50	10.7 ± 2.5	1.3	135 ± 8 (+15)

^a ^Based on approximately 100 grains; ^b^ ( ) Brackets indicate % change with respect to corresponding result of monolithic alloy.

X-ray diffraction (XRD) analysis revealed the presence of Al_12_Mg_17_ phase in the AZ31 based materials, and MgZn phase in the ZK60A based materials [21]. Al_2_O_3_ nanoparticle reinforcement as well as fine intermetallic distribution in the nanocomposites was reasonably uniform as shown in Figure 1. The reasonably uniform distribution of Al_2_O_3_ nanoparticles can be attributed to: (a) minimal gravity-associated segregation due to judicious selection of stirring parameters [22]; (b) good wetting of Al_2_O_3_ nanoparticles by the alloy matrix [23,24,25]; (c) argon gas disintegration of metallic stream [26]; and (d) dynamic deposition of composite slurry on substrate followed by hot extrusion. Selective area electron diffraction (SAED) in TEM mode revealed the partial reaction of Al_2_O_3_ nanoparticles with the magnesium alloy matrix to form MgO (where Al goes into solid solution and/or forms Mg-Al intermetallics) in each nanocomposite as shown in Figure 1 and listed in Table 2.

**Table 2:** Phase detection results of nanocomposites and nanoparticle reinforcement based on selected area electron diffraction (TEM).

k space (nm^−1^)	d (Å)	Phase	Plane	Reference d (Å)	JCPDS card #
AZ31/1.5 vol.% Al_2_O_3_ nanocomposite					
34.80	1.806	Al_12_Mg_17_	(5 3 0)	1.810	011128
**39.10**	**1.607**	**Al_2_O_3_**	**(1 1 6)**	**1.602**	**431484**
39.39	1.595	Mg	(1 1 0)	1.605	350821
39.59	1.587	Al_12_Mg_17_	(6 2 2)	1.600	011128
42.48	1.479	Mg	(1 0 3)	1.473	350821
49.70	1.264	Al_12_Mg_17_	(6 6 0)	1.250	011128
**64.69**	**0.971**	**MgO**	**(3 3 1)**	**0.966**	**450946**
65.66	0.957	Mg	(2 0 4)	0.951	350821
ZK60A/1.5 vol.% Al_2_O_3_ nanocomposite					
37.37	1.682	MgZn_2_	(2 1 1)	1.677	340457
39.39	1.595	Mg	(1 1 0)	1.605	350821
**40.55**	**1.549**	**Al_2_O_3_**	**(2 1 3)**	**1.553**	**260031**
**64.69**	**0.971**	**MgO**	**(3 3 1)**	**0.966**	**450946**
65.66	0.957	Mg	(2 0 4)	0.951	350821
67.60	0.930	MgZn_2_	(4 1 3)	0.932	340457
71.16	0.883	Mg	(3 0 2)	0.873	350821
Al_2_O_3_ nanoparticle reinforcement					
36.00	1.745	Al_2_O_3_	(0 2 4)	1.740	431484
40.55	1.549	Al_2_O_3_	(2 1 3)	1.553	260031
49.30	1.275	Al_2_O_3_	(2 0 8)	1.276	431484
69.30	0.907	Al_2_O_3_	(3 2 4)	0.908	431484

**Figure 2:**
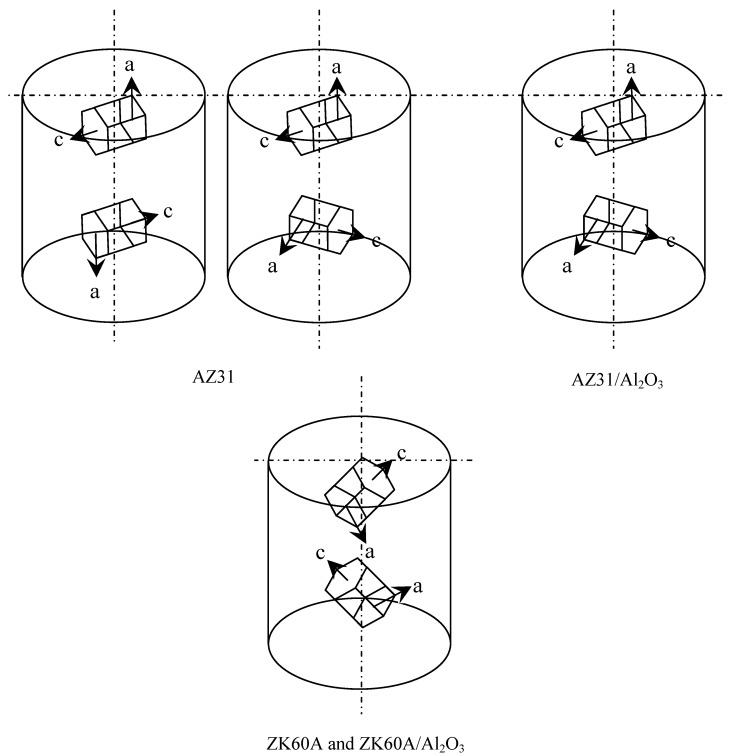
Schematic diagram showing textures of: (**a**) monolithic AZ31, (**b**) AZ31/Al_2_O_3_ nanocomposite and (**c**) ZK60A and ZK60A/Al_2_O_3_ nanocomposite based on X-ray diffraction. In each case, vertical axis is parallel to extrusion direction. Each cell is made up of 2 HCP units having 1 common (0 0 0 2) basal plane.

Texture results are listed in Table 3 and shown in Figure 2. In monolithic AZ31, the dominant textures in the transverse and longitudinal directions were (1 0 −1 0) and (1 0 −1 1) [and (0 0 0 2)], respectively. In the AZ31/Al_2_O_3_ nanocomposite, the dominant textures in the transverse and longitudinal directions were (1 0 −1 0) and (1 0 −1 1), respectively. Unlike monolithic AZ31, the AZ31/Al_2_O_3_ nanocomposite did not exhibit (0 0 0 2) dominant texture in the longitudinal direction. In monolithic ZK60A and derived nanocomposite, the dominant texture in the transverse and longitudinal directions was (1 0 −1 1).

**Table 3:** Texture results of AZ31, ZK60A and derived nanocomposites based on X-ray diffraction (goniometer).

Material	Section ^a^	Plane	Average I/I_max_^b^
AZ31	T	1 0 −1 0 prism	**1.00**
		0 0 0 2 basal	0.16
		1 0 −1 1 pyramidal	0.81
	L	1 0 −1 0 prism	0.27
		0 0 0 2 basal	**0.93**
		1 0 −1 1 pyramidal	**1.00**
AZ31/1.5 vol.% Al_2_O_3_	T	1 0 −1 0 prism	**1.00**
		0 0 0 2 basal	0.18
		1 0 −1 1 pyramidal	0.72
	L	1 0 −1 0 prism	0.23
		0 0 0 2 basal	0.64
		1 0 −1 1 pyramidal	**1.00**
ZK60A	T	1 0 −1 0 prism	0.12
		0 0 0 2 basal	0.25
		1 0 −1 1 pyramidal	**1.00**
	L	1 0 −1 0 prism	0.24
		0 0 0 2 basal	0.77
		1 0 −1 1 pyramidal	**1.00**
ZK60A/1.5 vol.% Al_2_O_3_	T	1 0 −1 0 prism	0.26
		0 0 0 2 basal	0.12
		1 0 −1 1 pyramidal	**1.00**
	L	1 0 −1 0 prism	0.30
		0 0 0 2 basal	0.48
		1 0 −1 1 pyramidal	**1.00**

^a^ T: transverse, L: longitudinal; ^b^ I_max_ is XRD maximum intensity from either prism, basal or pyramidal planes.

### 2.3. Hardness

The results of microhardness measurements are listed in Table 1. Each nanocomposite exhibited higher hardness than the corresponding monolithic alloy (by 30% and 15% for the AZ31/Al_2_O_3_ and ZK60A/Al_2_O_3_ nanocomposites, respectively). This was consistent with earlier observations made on Mg/Al_2_O_3_, AZ31/C_60_ and AZ31/MWCNT nanocomposites [14,27,28]. The increase in hardness of the nanocomposites in the present study can be attributed to: (a) reasonably uniform distribution of harder Al_2_O_3_ nanoparticles in the matrix and (b) higher constraint to localized matrix deformation during indentation due to the presence of nanoparticles [14,15,27].

### 2.4. Tensile Behavior

**Figure 3:**
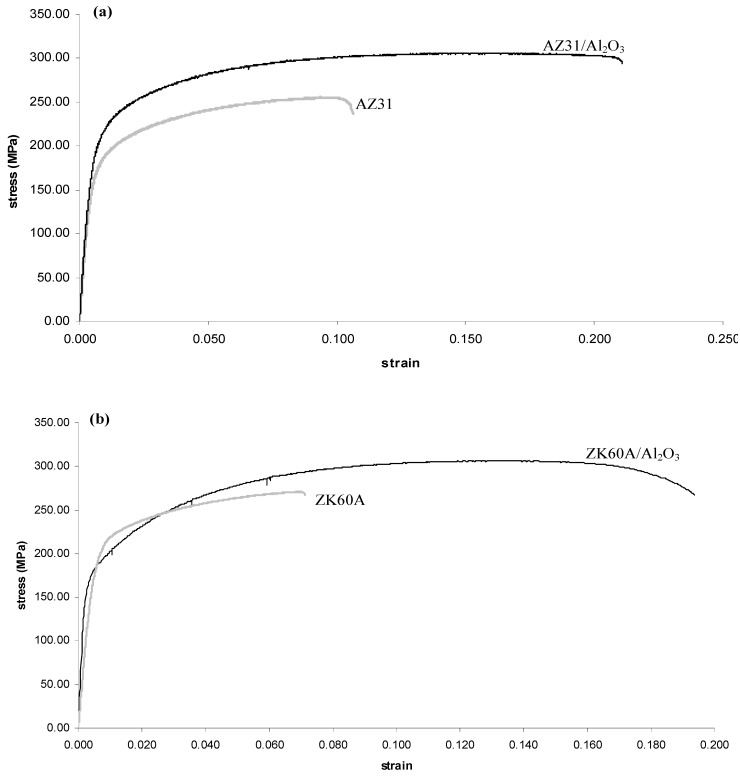
Representative tensile stress-strain curves of: (**a**) monolithic AZ31 and AZ31/Al_2_O_3_ nanocomposite; and (**b**) monolithic ZK60A and ZK60A/Al_2_O_3_ nanocomposite.

The overall results of ambient temperature tensile testing of the extruded materials are shown in Table 4 and Figure 3. The yield strength, ultimate strength, failure strain and work of fracture (WOF) of AZ31/Al_2_O_3_ nanocomposite were each significantly higher compared to monolithic alloy (by 19%, 21%, 113% and 162%, respectively). The WOF was determined by computing the area under the stress-strain curve. In the case of ZK60A/Al_2_O_3_ nanocomposite, the yield strength was decreased (by 4%) compared to monolithic alloy, while ultimate strength, failure strain and WOF were each significantly increased (by 13%, 170% and 200%, respectively).

**Table 4:** Results of tensile testing of AZ31, ZK60A and derived nanocomposites.

Material	0.2% TYS (MPa)	UTS (MPa)	Failure Strain (%)	WOF (MJ/m^3^) ^a^
AZ31	172 ± 15	263 ± 12	10.4 ± 3.9	26 ± 9
AZ31/1.5 vol.% Al_2_O_3_	204 ± 8 (+19) ^b^	317 ± 5 (+21)	22.2 ± 2.4 (+113)	68 ± 7 (+162)
ZK60A	182 ± 4	271 ± 1	6.7 ± 1.0	17 ± 3
ZK60A/1.5 vol.% Al_2_O_3_	175 ± 2 (−4)	305 ± 2 (+13)	18.1 ± 0.9 (+170)	51 ± 3 (+200)

^a^ Work of fracture, *i.e.*, area under the engineering stress-strain curve until the point of fracture (obtained using EXCEL software); ^b ^( ) Brackets indicate % change with respect to corresponding result of monolithic alloy.

Given the limited slip system activation in the HCP unit cell based structure of the AZ31 matrix at room temperature, the enhancement of tensile strength in the AZ31/Al_2_O_3_ nanocomposite can be first attributed to crystallographic texture difference between the nanocomposite matrix and monolithic alloy. In comparison of crystallographic texture, monolithic AZ31 exhibited (0 0 0 2) dominant texture in the longitudinal direction as listed in Table 3 and shown in Figure 2, unlike the AZ31/Al_2_O_3_ nanocomposite. For this (0 0 0 2) basal plane texture, basal slip is made most difficult due to the high critical resolved shear stress (CRSS) for slip based on the 0° angle between the (0 0 0 2) basal plane and the vertical axis as shown in Figure 2 [29,30]. However, non-basal slip was activated during deformation at room temperature due to basal plane alignment along the vertical (force) axis [31]. The tensile strength increase in the AZ31/Al_2_O_3_ nanocomposite compared to monolithic AZ31 can be next attributed to better known factors (pertaining to reinforcement) such as: (a) dislocation generation due to elastic modulus mismatch and coefficient of thermal expansion mismatch between the matrix and reinforcement [14,15,32,33]; (b) Orowan strengthening mechanism [32,33]; and (c) load transfer from matrix to reinforcement [14,32]. The same supporting attributes apply to the increase (by 13%) of ultimate strength in the ZK60A/Al_2_O_3_ nanocomposite compared to monolithic alloy. In the case of ZK60A/Al_2_O_3_ nanocomposite, the yield strength was decreased (by 4%) compared to monolithic alloy. This can be attributed to relatively increased leaching of Zr from the ZK60A matrix by Al_2_O_3_ nanoparticles [34].

In each nanocomposite, the failure strain increase compared to monolithic alloy can be attributed to the presence and reasonably uniform distribution of ceramic nanoparticles [27,35]. It has been shown in previous studies that the nanoparticles provide sites where cleavage cracks are opened ahead of the advancing crack front. This: (1) dissipates the stress concentration which would otherwise exist at the crack front; and (2) alters the local effective stress state from plane strain to plane stress in the neighborhood of crack tip [27,35]. WOF quantified the ability of the material to absorb energy up to fracture under load [36]. In each nanocomposite, the significantly increased WOF compared to monolithic alloy show the potential for using the nanocomposite in damage tolerant design.

### 2.5. Compressive Behavior

**Figure 4:**
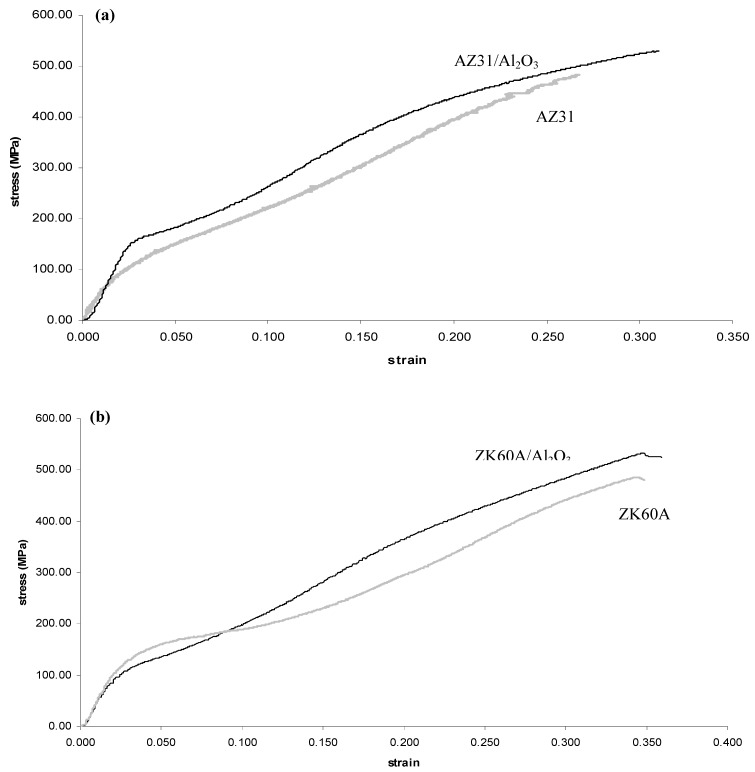
Representative compressive stress-strain curves of: (**a**) monolithic AZ31 and AZ31/Al_2_O_3_ nanocomposite; and (**b**) monolithic ZK60A and ZK60A/Al_2_O_3_ nanocomposite.

The overall results of ambient temperature compressive testing of the extruded materials are shown in Table 5 and Figure 4. The yield strength, ultimate strength and WOF of the AZ31/Al_2_O_3_ nanocomposite were each higher compared to monolithic alloy (by 5%, 5% and 11%, respectively), while failure strain of the AZ31/Al_2_O_3_ nanocomposite was not significantly decreased (by 4%) compared to monolithic alloy. In the case of the ZK60A/Al_2_O_3_ nanocomposite, the yield strength was decreased (by 10%) compared to monolithic alloy, while ultimate strength, failure strain and WOF were each increased (by 7%, 15% and 26%, respectively).

**Table 5:** Results of compressive testing of AZ31, ZK60A and derived nanocomposites.

Material	0.2% CYS (MPa)	UCS (MPa)	Failure Strain (%)	Work of Fracture, WOF (MJ/m^3^) ^a^
AZ31	93 ± 9	486 ± 4	19.7 ± 7.2	76 ± 14
AZ31/1.5 vol.% Al_2_O_3_	98 ± 2 (+5)^b^	509 ± 12 (+5)	19.0 ± 2.7 (−4)	84 ± 15 (+11)
ZK60A	93 ± 8	498 ± 16	23.2 ± 4.6	89 ± 12
ZK60A/1.5 vol.% Al_2_O_3_	84 ± 7 (−10)	532 ± 13 (+7)	26.7 ± 0.3 (+15)	112 ± 3 (+26)

^a^ Work of fracture, *i.e.*, area under the engineering stress-strain curve until the point of fracture (obtained using EXCEL software); ^b^ ( ) Brackets indicate % change with respect to corresponding result of monolithic alloy.

In each nanocomposite, the increase in strength can be attributed to similar better known factors (pertaining to reinforcement) as listed earlier regarding tensile strength increase. In the case of AZ31/Al_2_O_3_ nanocomposite, the increase in strength can be attributed to the nanocomposite not exhibiting (0 0 0 2) dominant texture in the longitudinal direction (unlike monolithic AZ31) as discussed earlier regarding tensile strength increase. In the case of the ZK60A/Al_2_O_3_ nanocomposite, the yield strength was decreased (by 10%) compared to monolithic alloy. This can again be attributed to the relatively increased leaching of Zr from the ZK60A matrix by Al_2_O_3_ nanoparticles [34].

In the case of the ZK60A/Al_2_O_3_ nanocomposite, failure strain increase compared to monolithic alloy can be attributed to the same reason as listed for tensile failure strain increase. In each nanocomposite, the increased WOF compared to monolithic alloy show the potential for using the nanocomposite in damage tolerant design.

### 2.6. Tensile/Compressive Yield Strength Anisotropy

In both the AZ31/Al_2_O_3_ nanocomposite and monolithic AZ31, 0.2% TYS was about double the 0.2% CYS (2.08 and 1.85, respectively, where yield strength anisotropy or YSA = 0.2% TYS/0.2% CYS). This can be attributed generally to {1 0 1 −2} < 1 0 1 −1 > -type twinning being more easily activated along the c-axis (of the HCP unit cell in Figure 2) during c-axis tension rather than c-axis compression [37,38]. With the c-axis of the HCP unit cell oriented 90° to the force axis (extrusion direction) in each case, {1 0 1 −2} < 1 0 1 −1 > -type twinning was less easily activated during tensile testing, when the c-axis was under compression.

In both the ZK60A/Al_2_O_3_ nanocomposite and monolithic ZK60A, 0.2% TYS was about 1.99 and 1.96 times (almost double) the 0.2% CYS, respectively. Here, the tensile/compressive YSA was approximately 2 despite the common crystallographic texture exhibited where {1 0 1 −2} < 1 0 1 −1 > -type twinning was activated along the c-axis of the hexagonal close packed (HCP) unit cell in Figure 2 with comparatively similar ease in both tension and compression along the c-axis, based on the 45° angle between the c-axis and the vertical axis [37,38]. Here, the tensile/compressive yield stress anisotropy can be attributed generally to half the strain rate used (less strain hardening) in compressive testing compared to tensile testing.

## 3. Experimental Section

### 3.1. Materials

In this study, AZ31 (nominally 2.50–3.50 wt.% Al, 0.60–1.40 wt.% Zn, 0.15–0.40 wt.% Mn, 0.10 wt.% Si, 0.05 wt.% Cu, 0.01 wt.% Fe, 0.01 wt.% Ni, balance Mg) rod supplied by Asianovena Pte Ltd (Singapore) and ZK60A (nominally 4.80–6.20 wt.% Zn, 0.45 wt.% Zr, balance Mg) supplied by Tokyo Magnesium Co. Ltd. (Yokohama, Japan) were used as the matrix material. AZ31 rod and ZK60A block were sectioned to smaller pieces. All oxide and scale surfaces were removed using machining. All surfaces were washed with ethanol after machining. Al_2_O_3_ nanopowder (50 nm size) supplied by Baikowski (Japan) was used as the reinforcement phase.

### 3.2. Processing

Monolithic AZ31 and ZK60A were cast using the disintegrated melt deposition (DMD) method [21,39]. This involved heating AZ31 or ZK60A sections to 750 °C in an inert Ar gas atmosphere in a graphite crucible using a resistance heating furnace. The crucible was equipped with an arrangement for bottom pouring. Upon reaching the superheat temperature, the melt was stirred for 2.5 min at 460 rpm using a twin blade (pitch 45°) mild steel impeller to facilitate the uniform distribution of heat. The impeller was coated with Zirtex 25 (86% ZrO_2_, 8.8% Y_2_O_3_, 3.6% SiO_2_, 1.2% K_2_O and Na_2_O, and 0.3% trace inorganics) to avoid iron contamination of the molten metal. The melt was then released through a 10 mm diameter orifice at the base of the crucible. The melt was disintegrated by two jets of argon gas oriented normal to the melt stream and located 265 mm from the melt pouring point. The argon gas flow rate was maintained at 25l pm. The disintegrated melt was subsequently deposited onto a metallic substrate located 500 mm from the disintegration point. Ingots of 40 mm diameter were obtained following the deposition stage. To form the AZ31/Al_2_O_3_ and ZK60A/Al_2_O_3_ nanocomposites, Al_2_O_3_ nanoparticle powder was isolated by wrapping in Al foil of minimal weight (<0.50 wt.% with respect to AZ31 or ZK60A matrix weight) and arranged on top of the AZ31 or ZK60A blocks, with all other DMD parameters unchanged. All ingots were sectioned into billets. All billets were machined to 36 mm diameter and hot extruded using 20.25:1 extrusion ratio on a 150 ton hydraulic press. The extrusion temperature was 350 °C. The billets were held at 400 °C for 60 min in a furnace prior to extrusion. Colloidal graphite was used as a lubricant. Rods of 8 mm were obtained. 

### 3.3. Microstructural Characterization

Microstructural characterization studies were conducted on metallographically polished monolithic and nanocomposite extruded samples to determine grain characteristics. Hitachi S4300 Field-Emission SEM was used. Image analysis using Scion software was carried out to determine the grain characteristics. Thin foils were prepared from the nanocomposite extruded samples using disc punch-out and ion-milling for (regarding localized effects): (a) nanoparticle distribution as well as (b) nanoparticle-matrix reactivity observation (based on selected area electron diffraction (SAED)) using transmission electron microscopy (JEOL JEM 3010 TEM with 300KeV accelerating voltage). Regarding SAED, nanopowder samples of Al_2_O_3_ dispersed in ethanol were also prepared by droplet application onto holey carbon film mounted on copper grids followed by drying. All k-space measurements (k) from SAED patterns were manually obtained and converted to crystallographic lattice d-spacings (d) based on d = 2π/k. Goniometer XRD studies were conducted using CuK_α_ radiation (λ = 1.5406 Å) with a scan speed of 2°/min in an automated Shimadzu LAB-X XRD-6000 diffractometer to determine intermetallic phase (s) presence and dominant textures in the transverse and longitudinal (extrusion) directions (regarding globalised effects). All d-spacings from SAED and goniometer XRD analysis were matched with corresponding d-spacings in the JCPDS database available in the Shimadzu LAB-X XRD-6000 diffractometer operating software to determine all phases present.

### 3.4. Hardness

Microhardness measurements were made on polished monolithic and nanocomposite extruded samples. Vickers microhardness was measured with an automatic digital Shimadzu HMV Microhardness Tester using 25 gf-indenting load and 15 s dwell time.

### 3.5. Tensile Testing

Smooth bar tensile properties of the monolithic and nanocomposite extruded samples were determined based on ASTM E8M-05. Round tension test samples of 5 mm diameter and 25 mm gauge length were subjected to tension using a MTS 810 machine equipped with an axial extensometer with a crosshead speed set at 0.254 mm/min.

### 3.6. Compressive Testing

Compressive properties of the monolithic and nanocomposite extruded samples were determined based on ASTM E9-89a. Samples of 8 mm length (l) and 8 mm diameter (d) where l/d = 1 were subjected to compression using a MTS 810 machine with 0.005 min^−1^ strain rate.

## 4. Conclusions

### 4.1. AZ31/Al_2_O_3_ Nanocomposite

Monolithic AZ31 and the AZ31/Al_2_O_3_ nanocomposite can be successfully synthesized using the DMD technique followed by hot extrusion.

Compared to monolithic AZ31, tensile strength of the AZ31/Al_2_O_3_ nanocomposite was enhanced. Compared to monolithic AZ31, compressive strength of the AZ31/Al_2_O_3_ nanocomposite was slightly increased. This can be commonly attributed to: (a) AZ31/Al_2_O_3_ nanocomposite not exhibiting (0 0 0 2) dominant texture in the longitudinal direction (unlike monolithic AZ31); and (b) well known factors pertaining to reinforcement. 

Compared to monolithic AZ31, tensile failure strain of the AZ31/Al_2_O_3_ nanocomposite was enhanced and the compressive failure strain was similar. This can be commonly attributed to reasonably uniform distribution of Al_2_O_3_ nanoparticles in the AZ31/Al_2_O_3_ nanocomposite.

Compared to monolithic AZ31, the AZ31/Al_2_O_3_ nanocomposite exhibited significantly high increment in tensile WOF and increment in compressive WOF.

### 4.2. ZK60A/Al_2_O_3_ Nanocomposite

Monolithic ZK60A and the ZK60A/Al_2_O_3_ nanocomposite can be successfully synthesized using the DMD technique followed by hot extrusion.

Compared to monolithic ZK60A, yield and ultimate strengths (in tension and compression) of the ZK60A/Al_2_O_3_ nanocomposite were decreased and increased, respectively. The decrease in yield strength can be attributed to the relatively increased leaching of Zr from the ZK60A matrix by Al_2_O_3_ nanoparticles. The increase in ultimate strength can be attributed to well known factors pertaining to reinforcement. 

Compared to monolithic ZK60A, tensile and compressive failure strain of the ZK60A/Al_2_O_3_ nanocomposite was significantly enhanced and enhanced, respectively. This can be attributed to the presence and reasonably uniform distribution of Al_2_O_3_ nanoparticles.

Compared to monolithic ZK60A, the ZK60A/Al_2_O_3_ nanocomposite exhibited significantly high increment in tensile WOF and increment in compressive WOF.
